# Life history shifts in an exploited African fish following invasion by a castrating parasite

**DOI:** 10.1002/ece3.6917

**Published:** 2020-10-29

**Authors:** Nestory Peter Gabagambi, Arne Skorping, Mwita Chacha, Kwendwa Jonathan Kihedu, Adele Mennerat

**Affiliations:** ^1^ Department of Biological Sciences University of Bergen Bergen Norway; ^2^ Department of Aquatic Sciences and Fisheries Technology College of Agricultural Sciences and Fisheries Technology University of Dar es Salaam Dar es Salaam Tanzania; ^3^ Tanzania Fisheries Research Institute Kyela Center Mbeya Tanzania

**Keywords:** African Great Lakes, environmental change, Lake Malawi sardine, Lake Nyasa, life history evolution, parasite invasion, Usipa

## Abstract

Evolutionary theory predicts that infection by a parasite that reduces future host survival or fecundity should select for increased investment in current reproduction. In this study, we use the cestode *Ligula intestinalis* and its intermediate fish host *Engraulicypris sardella* in Wissman Bay, Lake Nyasa (Tanzania), as a model system. Using data about infection of *E. sardella* fish hosts by *L. intestinalis* collected for a period of 10 years, we explored whether parasite infection affects the fecundity of the fish host *E. sardella*, and whether host reproductive investment has increased at the expense of somatic growth. We found that *L. intestinalis* had a strong negative effect on the fecundity of its intermediate fish host. For the noninfected fish, we observed an increase in relative gonadal weight at maturity over the study period, while size at maturity decreased. These findings suggest that the life history of *E. sardella* has been shifting toward earlier reproduction. Further studies are warranted to assess whether these changes reflect plastic or evolutionary responses. We also discuss the interaction between parasite and fishery‐mediated selection as a possible explanation for the decline of *E. sardella* stock in the lake.

## Graphical abstract

We studied the effect of the tapeworm *Ligula intestinalis* on the life history traits of its intermediate host *Engraulicypris sardella* in the African Great Lakes of Nyasa. We suggested that the life history of *E. sardella* has been shifting toward earlier reproduction due to infection of *L. intestinalis*.

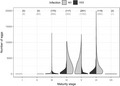

## 1 INTRODUCTION

Life history theory assumes that there are trade‐offs between different traits in organisms, such as growth, reproduction, and survival (Roff, 2002). These traits cannot be simultaneously maximized within the same individual because the available amount of nutrients and other resources are in limited supply (Stearns, 1989). Increased resource allocation into one trait will, therefore, come at the cost of reduced allocation into other traits (Agnew et al., 2000). In each given environment, the optimal way to resolve these trade‐offs (i.e., the optimal strategy for maximizing fitness) is the one achieving the highest possible reproductive success (Agnew et al., 2000; Pianka, 1976; Stearns, 1989). For instance, if adult mortality increases within a population (e.g., due to increased predation), individuals that mature relatively earlier and invest relatively more into current reproduction versus future survival will be favored by natural selection (Fredensborg & Poulin, 2006).

For fish, both natural predation and fishing (i.e., predation by humans) are important selective factors that drive adaptive changes in life history traits such as developmental rates and timing of reproduction (Heino & Godø, 2002; Jorgensen et al., 2007; Jørgensen et al., 2009; Sharpe et al., 2012). Fishing practices and predation are usually nonrandom factors, as gears are often designed to selectively take larger and older fish in the population (Law, 2000). In this case, smaller fish are likely to have a higher probability of survival than the larger ones, and among them, those that can mature and reproduce early will be selected (Jorgensen et al., 2007; Jørgensen et al., 2009). Assuming that early maturation is heritable to some extent, this should result in life histories changing toward earlier reproduction at smaller sizes (Ayllon et al., 2015; Heath et al., 2002; Olsen et al., 2004; Sinclair‐Waters et al., 2020).

Parasitism can also affect the future reproductive success of hosts (Fredensborg & Poulin, 2006) and thus select for changes in host life history traits (Adamo, 1999; Agnew et al., 1999; Lafferty, 1993b; McCurdy et al., 1999; Perrin et al., 1996; Polak & Starmer, 1998; Richner & Tripet, 1999; Sorci et al., 1996; Thomas et al., 2000; Yan et al., 1997). For instance, an increase in the prevalence of parasites causing castration (i.e., destruction or alteration of the host's gonadal tissues by the parasite (Noble & Noble, 1971)) can select for earlier maturity (Fredensborg & Poulin, 2006; Lafferty, 1993a; Loot et al., 2002; Minchella & Loverde, 1981). For the infected host, achieving reproduction prior to castration yields clear fitness benefits (Gooderham & Schulte‐Hostedde, 2011; Lafferty, 1993a; Minchella & Loverde, 1981), and these benefits increase along with infection risk (Minchella & Loverde, 1981; Polak & Starmer, 1998; Sorci et al., 1996). Increased reproductive effort in hosts exposed to castrating parasites has been reported in a number of species. So far, however, most documented life history changes seem to result from adaptive plastic responses of hosts to parasitic exposure, more than life history evolution following a change in parasite‐mediated selection (Chadwick & Little, 2005; Hudson et al., 2019; Vale & Little, 2012).

In this study, we investigated whether the castrating parasitic cestode *Ligula intestinalis* was responsible for a life history change in the cyprinid fish *Engraulicypris sardella* in Lake Nyasa. We studied the freshwater fish *E. sardella*, which is the second intermediate host for the cestode *L. intestinalis*. *E. sardella* (Günther, 1868), locally known as Usipa or Lake Malawi sardine, is a small, slender, silvery, zooplanktivorous fish endemic to Lake Nyasa (Lowe‐McConnell, 1993; Rufli & Van Lissa, 1982) that occurs in shoals, which are widely distributed within the lake and found in both nearshore areas and offshore pelagic water, down to a depth of approximately 200 m (Maguza‐Tembo et al., 2009).

*Engraulicypris sardella* is an annual species, where hatchlings grow and age to reproduce and die in a yearly cycle (Iles, 1960), although some studies indicate that they can live longer (Rusuwa et al., 2014; Thompson & Bulirani, 1993). They have been reported to breed throughout the year but with bi‐annual recruitment peaks occurring during the wet season and dry season (Morioka & Kaunda, 2005; Rusuwa et al., 2014).

During early developmental stages, *E. sardella* feeds exclusively on phytoplankton and then switches to feeding on zooplankton upon reaching adulthood (Allison et al., 1996; Degnbol, 1982). *E. sardella* demonstrates a rapid growth rate and can attain a maximum total length of about 130 mm in a year (Thompson, 1996; Tweddle & Lewis, 1990). Males and females mature at a size of about 70 and 75 mm, respectively (Thompson & Allison, 1997; Thompson et al., 1996).

*Engraulicypris sardella* forms an important part of the food web of Lake Nyasa. The species is primary consumer of zooplankton (Degnbol, 1982; Konings, 1990) and an important prey for pelagic piscivorous fishes, particularly *Diplotaxodon* spp. and *Rhamphochromis* spp. (Allison et al., 1996), as well as piscivorous birds (Linn & Campbell, 1992). *E. sardella* is also of high commercial value, and for many decades, it has been the main animal protein source for most of the local human population (Manyungwa‐Pasani et al., 2017). However, recently it has been observed that these cyprinids are infected by the cestode *L. intestinalis*.

*Ligula intestinalis* (L. 1758) is a common and widespread cestode, that uses cyprinid fish as the second intermediate host (Dubinina, 1980; Kennedy, 1974). The parasite is trophically transmitted and has a complex life cycle involving two aquatic intermediate hosts, a planktonic copepod and a fish (Dubinina, 1980; Loot et al., 2001). It reaches sexual maturity in the abdominal cavity of piscivorous birds that are the final hosts (i.e., the hosts where parasite reproduction takes place) (Dubinina, 1980; Loot et al., 2001). In infected fish, the parasite is found filling the body cavity (Hoole et al., 2010). Higher infection rates are observed in larger and older *E. sardella* than in juvenile individuals (Msafiri et al., 2014; Rusuwa et al., 2014), which can partly be explained by diet shifts from phytoplankton to zooplankton as *E. sardella* reaches maturity.

The invasion of *L. intestinalis* in Lake Nyasa was first noted in the late 1990s during longline research surveys where a milkish white worm was found in the body cavity of the endemic pelagic cyprinid fish *E. Sardella* (Mwambungu et al., 1996). The worm was identified to be the tapeworm *Ligula intestinalis* (L.). This parasite is believed to be introduced into Lake Nyasa by migrating fish‐eating birds such as the White‐breasted cormorant (*Phalacrocorax carbo*), which is one of the most abundant fish‐eating birds in the Lake Nyasa basin (Linn & Campbell, 1992) and one of the final hosts of *L. intestinalis* (Loot et al., 2001; Rosen, 1920). In Lake Nyasa, this cestode has been increasingly reported since it was first noted by Mwambungu et al. (1996). *E. Sardella* appears to be the only species used as intermediate fish host (Gabagambi et al., 2019; Gabagambi & Skorping, 2018; Msafiri et al., 2014; Rusuwa et al., 2014) (Figure S1).

*Ligula intestinalis* is known to induce castration in several intermediate hosts (Cowx et al., 2008; Hoole et al., 2010; Kennedy et al., 2001; Loot et al., 2002; Wyatt & Kennedy, 1988) and has therefore been suggested to cause population crashes of its host (Burrough et al., 1979; Kennedy et al., 2001). This could sometimes lead to local extinction of the parasite in small ecosystems (Kennedy et al., 2001). Recent results, however, indicate that local extinction of this parasite is unlikely in Lake Nyasa due to spatial and temporal variations in transmission rates (Gabagambi & Skorping, 2018).

Under such conditions of recent invasion, we hypothesize that the cestode *L. intestinalis* should select for a shift in resource investment from somatic growth toward reproduction in its intermediate fish host *E. sardella*. Using data collected from 2005 to 2015 in the northern part of Lake Nyasa, we address the following three questions:

(i) What are the effects of *L. intestinalis* on the fecundity of *E. sardella*? (ii) has reproductive investment at maturity of *E. sardella* increased over time? and (iii) has the average size at maturity of *E. sardella* decreased?

We then further discuss the selective roles of parasitic invasion versus other environmental factors that may recently have changed in Lake Nyasa.

## 2 METHODS

### 2.1 Study area

**FIGURE 1:**
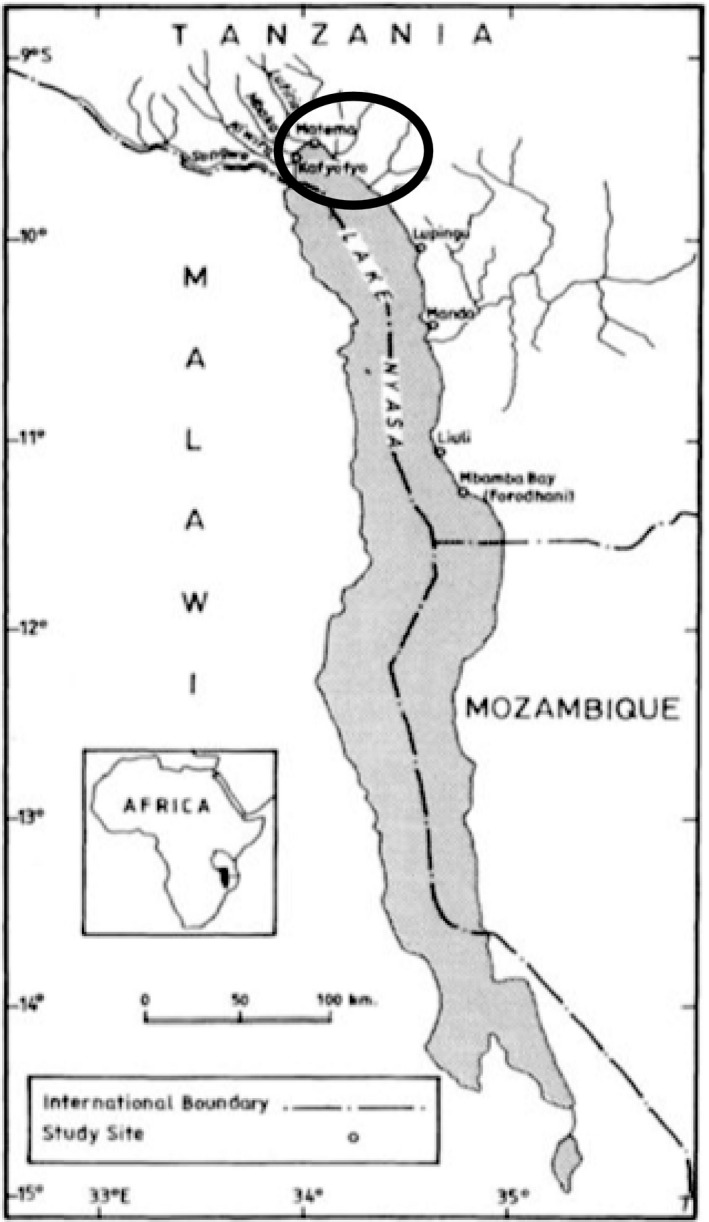
Map of Lake Nyasa showing fishing ground of Wissman Bay (in oval shape) Source: Modified from Msafiri et al. (2014).

The study was conducted in the northern part of Lake Nyasa, Tanzania side (Figure 1). Lake Nyasa, also known as Lake Malawi in Malawi and Lago Niassa in Mozambique, is the southernmost great lake in the East African Rift Valley system, located between Malawi, Mozambique, and Tanzania. The lake is the third largest freshwater lake in Africa after lakes Victoria and Tanganyika and is the second largest lake by volume after Lake Tanganyika (Darwall et al., 2010; Hampton et al., 2018; Macuiane et al., 2015). The lake has a maximum depth of 785 m, a volume of 8400 km^3^, a surface area of 29,000 km^2^, approximate length of 550 km, and mean width of around 48–60 km and is located 472 m above the sea level (Bootsma & Hecky, 1993; Darwall et al., 2010; Gonfiantini et al., 1979; Patterson & Kachinjika, 1995). The total catchment area of the lake is 126,500 km^2^ (Kumambala & Ervine, 2010) of which 97,750 km^2^ is land catchment (Menz, 1995). The mean surface temperature of the lake is between 24 and 28°C (Vollmer et al., 2005) and the annual rainfall ranges between 1000 and 2800 mm (LNBWB, 2013). The lake experiences two main seasons, the dry season (May–August) and wet season (November–April), which are governed by the regional climate (Lyons et al., 2011; Vollmer et al., 2005).

Lake Nyasa is meromictic, although it may experience mixing during the dry season in the southern tip of the lake where the depth is relatively shallow (Darwall et al., 2010; Vollmer et al., 2005; Weyl et al., 2010). Due to the stratification, together with the great depth of the lake, the nutrient availability to the plankton community are very low, and thus, the lake is considered “oligotrophic” (Irvine et al., 2001; Mwambungu & Ngatunga, 2001). The lake has more than 1,000 different fish species, many of which are endemic (Chafota et al., 2005; Salzburger et al., 2014). Sampling was conducted at Wissman Bay that is located at the northern end of the lake (sampling stations of Matema S9°29ʹ; E34°01ʹ, Mwaya S9°33ʹ; E33°57ʹ, Kafyofyo S9°35ʹ; E33°57ʹ, and Kiwira S9°37ʹ; E33°57ʹ).

### 2.2 Sampling procedure

Information on the infection of *E. sardella* fish hosts by the parasite *L. intestinalis* was collected over a period of 10 years, from 2005 to 2015 in Wissman Bay. No sampling was done in 2014 because the persons involved, and especially N.P. Gabagambi, needed to spend the year away pursuing studies and were not replaced. In the period 2005–2013, data were generated from fish caught by local fishermen from sites of Matema, Mwaya, Kafyofyo, and Kiwira within Lake Nyasa. *E. sardella* were caught using an open water seine net, locally known as “Ndaturu,” with 10 mm mesh size at a depth of about 100 m during the dark moon phase days. In 2015, fish were caught by our research team, using the same traditional fishing method as was used in the period 2005–2013.

The fishing procedure involved nine crew members using two dugout canoes and one large plank boat. On the fishing ground, one of the dugout canoes was equipped with pressurized paraffin lamps (between one and three) and was stationed with one crew member away from the remaining vessels. The artificial light was used to concentrate the fish into the given area. This process took several hours. After a sufficient amount of fish had been attracted, the other unlit fishing vessels simultaneously deployed a net in a semicircular shape around the concentrated fish, and this was hauled by hand into the plank boat. A total of 3488 female *E. sardella* were sampled (Table 1). Males were also caught and examined as part of general monitoring, but due to the low reliability of stage determination for males of such a small fish species, only females were included in this study.

**TABLE 1:** Numbers of *E. sardella* per maturity stage sampled at Wissman Bay, Lake Nyasa, between 2005 and 2013 and then in 2015

Year	Maturity stage							Total	Prevalence (%)
	I	II	III	IV	V	VI	VII		
2005			13 (6)	12 (6)	126 (64)			151 (76)	50
2006		1 (0)	195 (51)	40 (11)	38 (1)	337 (60)		611 (123)	20
2007			212 (8)	45 (6)	529 (140)	149 (37)		935 (191)	20
2008		2 (0)	59 (13)	14 (9)	6 (2)	153 (12)	3 (2)	237 (38)	16
2009			16 (7)	15 (6)	215 (8)	52 (2)		298 (23)	8
2010		14 (0)	47 (11)	14 (6)	31 (14)	17 (8)		123 (39)	32
2011		2 (0)	28 (10)	13 (5)	2 (1)	3 (0)		48 (16)	33
2012	4 (0)	17 (0)	136 (37)	23 (18)				180 (55)	31
2013	2 (0)	24 (0)	68 (19)	28 (17)	8 (3)			130 (39)	30
2015			62 (8)	215 (33)	498 (58)			775 (99)	13
Total	6 (0)	60 (0)	836 (170)	419 (117)	1453 (291)	711 (119)	3 (2)	3488 (699)	20
% of all stages	0.17	1.7	24	12	41.7	20.4	0.09		
Prevalence (%)	0	0	20	28	20	17	67	20	

Note

Number in parentheses show the number of infected fish out of the sampled fish.

Upon landing, the total length and weight of each *E. sardella* were measured to the nearest 5 mm and 0.01 g, respectively. Specimens of *E. Sardella* were kept in cool boxes until further examination. The fish were later dissected for parasite determination. *L. intestinalis* was identified according to the protocol by Dobben (1952) while examination of other parasites was done according to Parpena (1996). The sex of *E. sardella* was determined using a stereomicroscope (Wild Heerbrugg M5) at 6.4X magnification. Gonad maturity was assessed on a seven‐stage maturity scale (Table 2), modified from Holden and Raitt (1974).

**TABLE 2:** Gonad maturity stages of a female *E. sardella* modified from Holden and Raitt (1974)

Maturity stage	Maturity status	Maturity description
I	Immature	Immature fish with ovaries in a pinkish‐translucent color
II	Maturing	Maturing fish with ovaries in pinkish color
III	Ripening	Ripening fish with ovaries in pinkish‐yellow color
IV	Ripe	Prespawning fish with ovaries in orange‐pinkish color with conspicuous superficial blood vessels
V	Partial spent	Spawning fish with ripe ovaries
VI	Running	Ovaries yellowish‐brown
VII	Spent	Ovaries loose and flabby

For seven years of the ten years (i.e., 2005, 2006, 2010, 2011, 2012, 2013, and 2015), the maturity stages of *E. sardella* were determined and recorded by the same investigator (N.P. Gabagambi). Therefore, we were able to maintain a good level of consistency and accuracy in the determination of maturity stage across our sampling period. In 2007, 2008, and 2009, maturity determination was carried out by trained research technicians (E.J. Magesa and J.M. Masore), following the same seven‐stage maturity scale as was applied in all other sampling years.

Gonads were weighed to the nearest 0.01 g (wet weight) using sensitive precision balances (vwr™—model ECN 611–2315 and Endel™—model WPS) and fecundity for infected and noninfected female *E. sardella* was determined through gravimetric methods (Holden & Raitt, 1974) by counting the advanced yolked oocytes present in ripe and gravid *E. sardella*. The complete ovary was taken out and preserved in modified Gilson's fluid (100 ml 60% alcohol, 800 ml water, 15 ml 80% nitric acid, 18 ml glacial acetic acid, 20 g mercuric chloride) for 24 hr. Thereafter, the ovaries were shaken periodically to help loosen the eggs from connecting ovarian tissues. After the eggs were liberated from the ovarian tissues, they were washed thoroughly, spread on blotting paper, and allowed to dry at ambient temperature ranging between 25 and 30°C. Thereafter, the total numbers of eggs were weighed to the nearest 0.01 g using sensitive precision balance to have a total weight of eggs. Afterward, we collected a random subsample of the eggs, which were weighed and counted out on petri dish subsections using a stereomicroscope (Wild Heerbrugg M5) at 6.4× magnification. The total number of eggs (i.e., fecundity) in the ovaries was calculated following the formula given by Holden and Raitt (1974) as follows: *F* = *nG*/*g*, where *n* = number of eggs in subsample, *G* = total weight of eggs from the ovary, and *g* = weight of the subsample. Fish somatic weight was determined by subtracting the gonad weight from the total weight of the fish.

### 2.3 Statistical analyses

All statistics and graphics were carried out using R, version 3.2.5 (http://r‐project.org).

- The effect of *L. intestinalis* infection on host fecundity was tested using a generalized linear mixed‐effects model (glmmPQL) fitted with fecundity as a response variable (assuming quasi‐Poisson distribution), and maturity stage and infection status as predictor variables. Because the data were collected over a 10‐year period, year of sampling was included as a random effect factor in the model.
- To test whether reproductive investment at maturity has increased over time, we used a generalized linear model (glm) fitted with a binomial distribution. The binomial response variable combined gonadal weight of uninfected *E. sardella* and somatic weight. We chose to use relative gonad weight at stage IV because this is the stage where *E. sardella* reach reproductive maturity. Year was included as a numerical predictor variable.
- To test whether size of *E. sardella* at maturity has decreased over time, we first fitted for each year a logistic regression model with maturity status as a binomial response variable (0: immature; 1: mature) and body length as a continuous predictor variable (Figure S2). From the parameters of these logistic regression equations, and following Diaz Pauli and Heino (2013), we estimated for each year the length at which the probability of maturing is 50% (i.e., LM_50_):

$${\mathrm{LM}}_{50}=\frac{\mathrm{Log}e\left(\frac{p}{1‐p}\right)‐\left(a\right)}{b}$$

where *p* is the probability of maturity (0.5), *a* is the intercept, and *b* is the slope.

To test whether LM_50_ decreased over time, we fitted a linear model (lm) with LM_50_ as a response variable and year as a numerical predictor (linear and quadratic terms).

## 3 RESULTS

**FIGURE 2:**
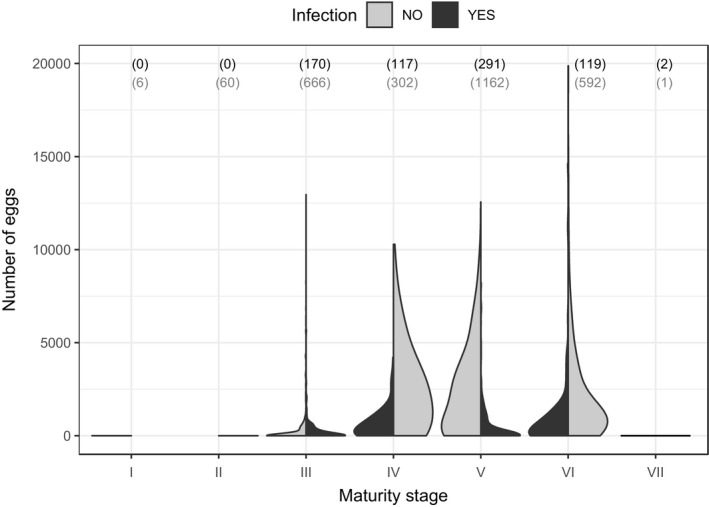
Fecundity (number of eggs in the gonads) of *E. sardella* at various maturity stages, for noninfected fish (gray) and fish infected by *L. intestinalis* (black). Both the distribution and probability density of data are represented here. Sample sizes are indicated in parentheses

A total of 3,488 individuals were sampled and measured for length, weight, gonad maturation, and fecundity over the study period (Table 1). Infected individuals had an overall lower fecundity than noninfected individuals (glmmPQL, estimate = −1.08 ± 0.08, *df* = 3416, *t* = −13.92, *p* < .001; Figure 2).

**FIGURE 3:**
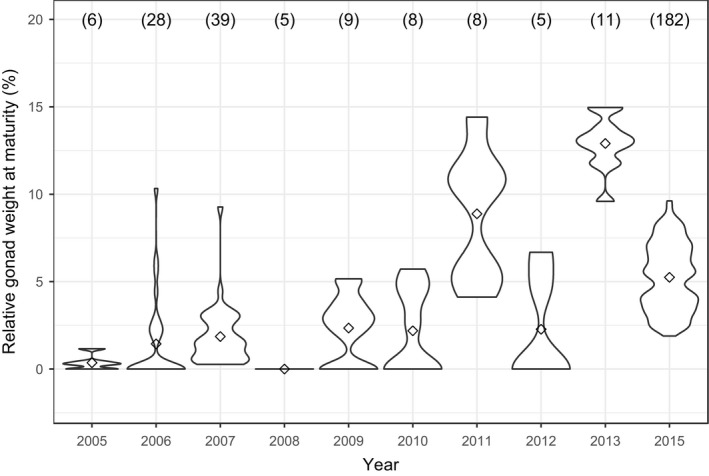
Temporal increase in reproductive investment at maturity (stage IV) of noninfected *E. sardella*. Both the distribution and probability density of data are represented here. Dots indicate mean values. Sample sizes are indicated in parentheses

Reproductive investment at maturity (relative weight of gonads at stage IV) in noninfected *E. sardella* increased significantly from 2005 to 2015 (glm, estimate = 0.14 ± 0.01, *df* = 1, *t* = 9.59, *p* < .001; Figure 3).

**FIGURE 4:**
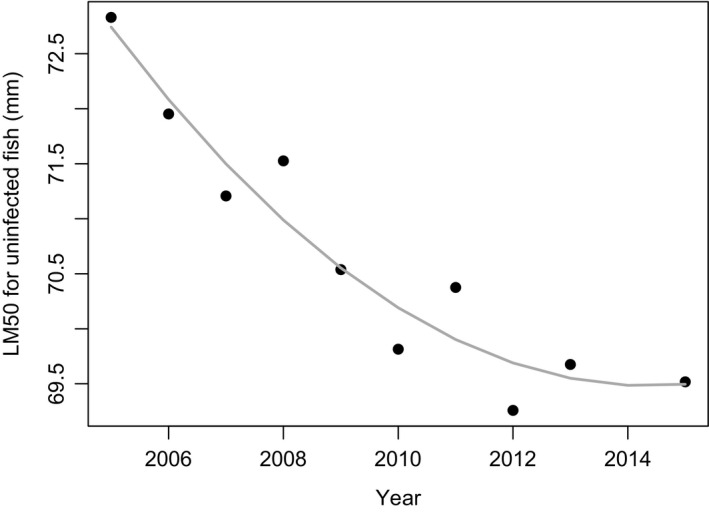
Temporal changes in LM_50_ for female *E. sardella*, *that is,* the estimated length at which the probability of maturing is 0.5 (final model represented by gray curve and estimated LM_50_ for each year by solid black dots)

To test whether LM_50_ (the length at which the probability of maturing is 0.5) varied over time, we fitted two models, one with and one without a quadratic term for year. The model with quadratic term was retained as final model due to lower residual deviance (null deviance = 12.48; residual deviance with quadratic term = 0.97; residual deviance without quadratic term = 2.09) and lower AIC value (AIC, with quadratic term = 13.01; without quadratic term = 18.71). LM_50_ decreased significantly over time (lm, year: estimate = −150 ± 52.5, *df* = 1, *t* = −2.86, *p* = .02; year^2^: estimate = 0.04 ± 0.01, *df* = 1, *t* = 2.85, *p* = .02; Figure 4).

## 4 DISCUSSION

*Ligula intestinalis* had a strong negative effect on the fecundity of its intermediate host, *E. sardella*. Such an effect, which was also found in other fish host species, thus seems widespread throughout the species range of this parasite (Barson & Marshall, 2003; Carter et al., 2005; Cowx et al., 2008). We also found that the relative weight of gonads increased, while body size at maturity decreased, over the 10‐year duration of this study. These temporal changes, found in noninfected fish, indicate that investment of *E. sardella* into early reproduction has increased at the expense of somatic growth.

This study took place a few years only after the arrival of *L. intestinalis* in the lake. A parasitic relationship between *L. intestinalis* and *E. sardella* in Lake Nyasa was indeed first observed in 1996 (Mwambungu et al., 1996). An earlier study investigating the breeding biology and in particular examining the ovaries of *E. sardella* between 1992 and 1994, did not report any case of *L. intestinalis* infection (Thompson, 1996). This tapeworm was thus likely absent from Lake Nyasa prior to the late 1990s. After the first observation, *E. sardella* in the lake kept being found infected by *L. intestinalis*, as manifested by the work of J.K. Kihedu (MSc thesis, Sokoine University of Agriculture, Tanzania, 2006, unpublished data). The earliest sampling year in our study is 2005, when prevalence is estimated at 50% (Table 1). This indicates that *L. intestinalis* had spread and therefore that the selection caused by this parasite on its host had increased steadily during the early years after introduction. Our study remains correlative, yet given the timing of the observed life history shift relative to the invasion of the lake by *L. intestinalis*, it seems legitimate to consider parasitism as a likely contributing factor.

In general, changes in age‐specific mortality or fecundity rates lead to changes in selection on life history traits. In our study, we observed an overall 69% lower fecundity in infected versus uninfected hosts, that is, the cestode *L. intestinalis* caused a significant partial castration in *E. sardella*. Reduced host fecundity is a common outcome of parasite infection (Gooderham & Schulte‐Hostedde, 2011; Hurd, 2001), but is especially severe for castrating parasites. Castration selects for higher, earlier reproductive effort, as those individuals that are able to reproduce before castration are clearly favored (Forbes, 1993). A number of host species have been shown to increase their early reproductive effort when parasitism reduces their chances for future reproduction (Adamo, 1999; Jokela & Lively, 1995; Lafferty, 1993b; Minchella & Loverde, 1981). This kind of adaptive response can result from two distinct mechanisms, namely plasticity or evolution, and distinguishing between the two can reveal challenging.

Plastic life history shifts toward increased investment in early reproduction in exposed and/or infected hosts have been reported for a range of host–parasite systems. In insects, Polak and Starmer (1998) observed that experimentally parasitized male *Drosophila nigrospiracula* infected with a mite (*Macrocheles subbadius*) lived shorter lives, but before dying they courted females significantly more than nonparasitized controls. Further, Adamo (1999) observed that female crickets (*Acheta domesticus*) increased egg laying in response to infection with the bacterium *Serratia marcescens*. In snails, Minchella and Loverde (1981) and Thornhill et al. (1986) observed an increase in reproductive output in female *Biophalaria glabrata* parasitized by a castrating trematode *Schistosoma mansoni*. In crustaceans, Chadwick and Little (2005) observed that *Daphnia magna* infected with a microsporidian *Glugoides intestinalis* shifted their life history toward early reproduction. In birds, Sanz et al. (2001) observed that female pied flycatchers (*Ficedula hypoleuca*) with hemoparasite infection initiated egg laying earlier and laid larger clutches. In reptiles, Sorci et al. (1996) observed that common lizards (*Lacerta vivipara*) increased their reproductive investment after being infected with haematozoans. More examples where reproduction is seen to increase with the onset of infection have been reviewed in Schwanz (2008). Taken together, these studies show that parasites, by affecting the future reproductive success of their hosts, can induce plastic life history changes in infected hosts that are adaptive.

Here, we observe a shift toward increased reproductive effort at the expense of somatic growth across generations. This pattern is found in noninfected hosts and therefore cannot be explained by plastic responses to infection. In addition, given the empirical evidence available at this stage, plastic responses to exposure appear unlikely, given the lack of clear correlation between yearly fluctuations in prevalence and life history trends, as one would expect under such a scenario. We therefore cannot exclude that our results may reflect adaptation to recent changes in Lake Nyasa.

Importantly, increased parasite pressure may not be the only environmental change that has taken place in Lake Nyasa over the last couple of decades and that might have triggered life history responses in *E. sardella*. Other potential sources of selection for earlier reproduction include fishing (Fenberg & Roy, 2008; Heino & Godø, 2002; Hutchings & Fraser, 2008; Jorgensen et al., 2007; Jørgensen et al., 2009; Kuparinen & Merilä, 2007; Sharpe & Hendry, 2009; Sharpe et al., 2012); increased predation by native or introduced species (Hampton et al., 2018; Sharpe et al., 2012); and fluctuations in zooplankton abundance that may induce earlier maturation.

Most evidence of fishery‐induced evolution comes from large, heavily exploited fish population stocks (e.g., North Arctic cod) where industrial fishing using trawlers has been in practice for many years. On the contrary, the Lake Nyasa *E. sardella* fishery is mainly traditional, operating in nearshore lake zones using paddled dugout canoe crafts (Mwambungu & Ngatunga, 2001). In the last years of this study, however, *E. sardella* stocks have collapsed, despite no sudden changes in fishing effort. As a consequence fishing pressure has dramatically increased in Wissman bay (Figure S3).

In the present study, *E. sardella* were sampled using the traditional fishing method. The majority of the sampled fish was composed of individuals of the body sizes between 50 and 100 mm in length, which corresponds to mature fish (i.e., from stage IV and above). This suggests that the traditional *E. sardella* fishing practice is probably size‐selective and induces a higher mortality in adults than younger fish, thus possibly reinforcing the selective effects of parasitism. Interestingly, the dramatic decrease in landings in 2013 was preceded by three consecutive years with high *L. intestinalis* prevalence (Figure S3), further suggesting that parasitism is a strong selective factor. In this system, *L. intestinalis* may have acted synergistically with fishery‐mediated selection in driving what appears like an evolutionary shift toward earlier reproduction of *E. sardella* in Lake Nyasa.

Increased predation by native or introduced organisms could also be one factor affecting selection on life history traits of *E. sardella*. In the native cyprinid fish *Rastrineobola argentea* in Napoleon Gulf of Lake Victoria, Sharpe et al. (2012) observed decreased body size, maturation at smaller sizes, and increased reproductive effort in response to the introduced predator fish *Lates niloticus*. However, in contrast to Lake Victoria and many other ancients lakes where dozens of non‐native species have been introduced over the past decade (Hampton et al., 2018), in Lake Nyasa no new introduced predator for *E. sardella* has been reported so far. The primary natural piscivorous predators of *E. sardella* in this lake are the pelagic haplochromine cichlids from the genera *Ramphochromis, Diplotaxodon,* and *Copadichromis,* as well as the larger cyprinids *Opsaridium microlepis* and *O. microcephalum*. Increased abundance of the native predators of *E. sardella* over time in the lake could have selected for life history changes similar to those observed here. Unfortunately, the area where the present study was conducted is a data‐poor region; the last pelagic ecosystem stock assessment was conducted between 1991 and 1994 (Menz (1995). Recent time series on abundance fluctuations of the natural predators of *E. sardella* are lacking. Further research, particularly on the combined effects of parasitism, fishing, and natural predation on *E. sardella* in Lake Nyasa, would be highly valuable, given the ecological and economical importance of this fish species.

Another factor that could have affected selection on the life history traits of *E. sardella* in Lake Nyasa may be parallel increases in the prevalence of other parasites. In their natural habitats, hosts are usually infected by two or more different parasite species (Kotob et al., 2017; Petney & Andrews, 1998). To the best of our knowledge, the only other parasite that has been reported to infect *E. sardella* is the nematode *Camallanus* sp. (Mgwede & Msiska, 2018). In the present study, we caught 3,488 wild, *that is,* naturally infected *E. sardella*, none of them observed with *Camallanus* sp. infection.

Overall, this study reveals that life history of *E. sardella* in Lake Nyasa has been shifting, over a period corresponding to the invasion of this lake by a castrating parasite. It is correlative, and the causative links between parasitism and life history changes remain to be established. Yet, the cestode *L. intestinalis*, by strongly reducing the fecundity of its host, appears as a likely driver of life history evolution, similar in its effects to size‐selective fisheries. In Lake Nyasa, these two types of selective factors may have acted concomitantly. More work is now warranted to examine the origin of these changes and determine whether they represent plastic or evolutionary responses.

## CONFLICT OF INTEREST

None declared.

## AUTHOR CONTRIBUTION

**Nestory Peter Gabagambi։** Writing original draft (lead); methodology‐data collection (lead). **Arne Skorping։** Conceptualization (supporting); review and editing (equal). **Mwita Chacha։** Review and editing (equal). **Kwendwa Jonathan Kihedu։** Review and editing (equal). **Adele Mennerat։** Conceptualization (lead); writing original draft (supporting); methodology‐statistical analysis (lead); review and editing (equal).

## ETHICS STATEMENT

This research received ethical approval from Tanzania Fisheries Research Institute (Application ID: TAFIRI/HQ/PF637/100).

### Open Research Badges

This article has earned an Open Data Badge for making publicly available the digitally‐shareable data necessary to reproduce the reported results. The data is available at https://doi.org/10.5061/dryad.p2ngf1vp3.

## Supporting information

Figures S1‐S3

## Data Availability

Data sets supporting this manuscript can be accessed through։ https://doi.org/10.5061/dryad.p2ngf1vp3.
