# Supplementary material for: Life history shifts in an exploited African fish following invasion by a castrating parasite
Source: Ecol Evol. 2020 Oct 29;10(23):13225–35. doi: 10.1002/ece3.6917 (PMC7713912; doi:10.1002/ece3.6917)
Supplement: Supplementary file 1 — Figures S1‐S3 [file ECE3-10-13225-s001.docx]

**Appendix A figures**

**Figure A1**


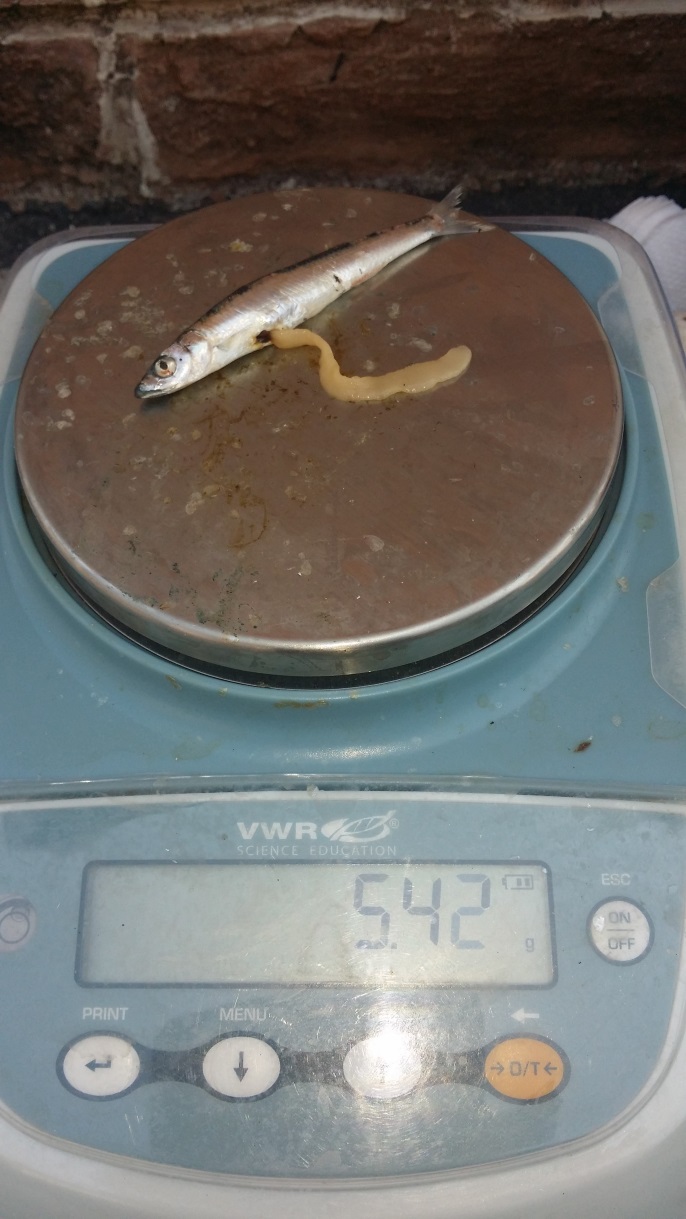


**Figure A2**

**Figure A3**

**Appendix A legends**

**Figure A1.** Laboratory picture of tapeworm *L. intestinalis* and its host fish *E. sardella*. Source: Nestory Peter Gabagambi (2015).

**Figure A2.** Logistic regression models were fitted for each year, and their parameters were used to estimate LM_50_ for each year (i.e., the age at which the probability of maturing is 0.5).

**Figure A3.** Yearly trends for LM50 (top panel), *L. intestinalis* prevalence in female *E. sardella* (upper middle), fishing pressure in Lake Nyasa, taken as the number of fishermen per tonne landed (lower middle), and *E. sardella* landings (bottom).Source from fisheries data: Kyela District Council, Department of Fisheries. Models represented by grey lines, data by black dots.
